# Training and research integration in an orthogeriatrics unit

**DOI:** 10.1590/S1516-31802007000300013

**Published:** 2007-05-03

**Authors:** Luiz Eugênio Garcez Leme

Physicians involved in surgery and correlated fields are increasingly being required to carry out interventions taking into account the specific needs of the elderly population. Since 1994, the American Geriatrics Society (AGS) has been running a project to improve the quantity and quality of education and training in geriatrics for residents in surgery and correlated fields. The Geriatrics Education for Specialty Residents (GESR) project includes specific training in 29 residency programs in different fields, including pilot methods designed to integrate geriatric knowledge into specialized medical residency programs. Initially, the disciplines chosen were: General Surgery, Emergencies, Gynecology, Orthopedics and Urology, followed by Physical Medicine, Rehabilitation and Thoracic Surgery.^1^

Just like other developing countries, Brazil is experiencing the dramatic situation of demographic aging.^2^ It is estimated that by 2025 the elderly population in Brazil will exceed 32 million.^3^ Representing 8.45% of the general population, the elderly in Brazil account for 19.4% of hospitalizations for all causes, 22% of orthopedic hospitalizations and 42% of hip fractures. The mortality rate among the population aged 60 and over is 2.9 times higher, with a cost per hospitalization 32% higher than for the population in general.^4^ In spite of the accelerated aging of its population, Brazil is out of step with more developed countries in terms of geriatric attention. Currently, less than 500 Brazilian physicians hold a specialization degree in Geriatrics, which means one geriatrician for every 37,000 elderly Brazilians.^2^

In 1996, in the Faculdade de Medicina da Universidade de São Paulo (FMUSP), where Geriatrics had been a regular discipline in the medical course since 1984, the Orthopedics Department started its Orthogeriatrics Group, with the specific purpose of providing specialized attention to elderly patients. From the very beginning, the group has promoted intense interdisciplinary and interprofessional guidance, working with a team of geriatricians, orthopedists, anesthetists, nurses, physiotherapists, social workers, nutritionists, psychologists, occupational therapists and speech therapists. The group provides differentiated attendance in geriatrics and orthopedics to patients aged 60 years and over in emergency rooms, wards, and pre and postoperative units, as well as outpatient attendance. It also provides home visits 10 to 14 days after hospital discharge, plus training for informal caregivers.^5^

The educational activities of the group, which are linked to the disciplines of both orthopedics and geriatrics in FMUSP, include an elective discipline of Orthogeriatrics in the undergraduate medical course and participation in the disciplines of General Orthopedics, Geriatrics and Orthopedic Emergencies in the third, fourth, and sixth years of the undergraduate medical course. In the first year of Orthopedics residency, residents spend one month in the geriatric care unit. Likewise, fellowship in Geriatrics also includes a stage in the Orthogeriatrics unit.

There has been a noticeable decrease in nosocomial morbidity and mortality among the elderly patients over the years of the group's existence. Furthermore, over these years, there has been a noticeable change among the orthopedic residents and their preceptors, manifested by more appropriate prescriptions and surgical indications for elderly patients. Likewise, geriatrics residents and fellows have developed new skills in terms of clinical support for orthopedic surgery. This association has been accompanied by a reduction in iatrogenic problems and development of a consistent interdisciplinary policy.^5^

In our opinion, this experience could give support to the mission of making surgeons, orthopedists and the entire non-gerontological health community more sensitive and better prepared to face our problems of demographic aging.
